# Multi-Slice Generation sMRI and fMRI for Autism Spectrum Disorder Diagnosis Using 3D-CNN and Vision Transformers

**DOI:** 10.3390/brainsci13111578

**Published:** 2023-11-10

**Authors:** Asrar G. Alharthi, Salha M. Alzahrani

**Affiliations:** Department of Computer Science, College of Computers and Information Technology, Taif University, Taif 21944, Saudi Arabia; asrar.g.alharthi@gmail.com

**Keywords:** autism spectrum disorder, ASD, fMRI, sMRI, neuroimaging, vision transformer, ConvNeXt, MobileNet, ViT, Swin, 3D-CNN

## Abstract

Researchers have explored various potential indicators of ASD, including changes in brain structure and activity, genetics, and immune system abnormalities, but no definitive indicator has been found yet. Therefore, this study aims to investigate ASD indicators using two types of magnetic resonance images (MRI), structural (sMRI) and functional (fMRI), and to address the issue of limited data availability. Transfer learning is a valuable technique when working with limited data, as it utilizes knowledge gained from a pre-trained model in a domain with abundant data. This study proposed the use of four vision transformers namely ConvNeXT, MobileNet, Swin, and ViT using sMRI modalities. The study also investigated the use of a 3D-CNN model with sMRI and fMRI modalities. Our experiments involved different methods of generating data and extracting slices from raw 3D sMRI and 4D fMRI scans along the axial, coronal, and sagittal brain planes. To evaluate our methods, we utilized a standard neuroimaging dataset called NYU from the ABIDE repository to classify ASD subjects from typical control subjects. The performance of our models was evaluated against several baselines including studies that implemented VGG and ResNet transfer learning models. Our experimental results validate the effectiveness of the proposed multi-slice generation with the 3D-CNN and transfer learning methods as they achieved state-of-the-art results. In particular, results from 50-middle slices from the fMRI and 3D-CNN showed a profound promise in ASD classifiability as it obtained a maximum accuracy of 0.8710 and F1-score of 0.8261 when using the mean of 4D images across the axial, coronal, and sagittal. Additionally, the use of the whole slices in fMRI except the beginnings and the ends of brain views helped to reduce irrelevant information and showed good performance of 0.8387 accuracy and 0.7727 F1-score. Lastly, the transfer learning with the ConvNeXt model achieved results higher than other transformers when using 50-middle slices sMRI along the axial, coronal, and sagittal planes.

## 1. Introduction

Autism spectrum disorder (ASD) is a complex neurodevelopmental disorder that affects an individual’s social and communication skills, as well as their behavior and interests. Although the exact causes of autism spectrum disorder (ASD) are not fully understood, it is believed to be a combination of genetic and environmental factors. Identifying ASD at an early stage allows individuals to receive the necessary assistance at a crucial stage of their development, enabling them to overcome challenges and maximize their potential for growth and progress. Neuroimaging techniques, including magnetic resonance images, shortly MRI, have become valuable clinical tools for exploring the underlying neural mechanisms of brain disorders. While MRI offers numerous advantages, such as high-resolution imaging for detecting smaller anatomical structures and abnormalities, it comes with the drawback of generating an ever-increasing number of images per patient [1]. As the number of images increases, clinicians are faced with the challenge of interpreting them, and the workload associated with this task has the potential to overwhelm their ability to effectively interpret them. Additionally, MRI scans have different protocols and records with multiple slices, requiring clinicians to be very precise to thoroughly examine each slice [2]. Most clinicians find it difficult to use MRI images to diagnose ASD in its earlier stages. Recently, there has been a notable increase in research utilizing machine learning (ML) models alongside MRI modalities for ASD diagnosis. Most research works have focused on extracting features from MRI modalities for ASD diagnosis using ML [3,4,5,6,7,8,9,10,11]. Recent research studies have investigated the use of MRI modalities as input to deep learning (DL) models mainly convolutional neural networks (CNNs) [2,12,13,14,15,16,17,18,19,20,21,22,23,24,25,26], autoencoders [25,27,28,29], and transformer attention mechanisms [17,30,31,32,33,34]. One constraint observed in these studies that employed DL for automatic feature extraction and classification in ASD was the use of small datasets for training their models.

Transfer learning (TL) has gained popularity in recent years due to the success of deep learning in the field of computer vision [35]. It is an important method when dealing with a small amount of data, as it makes use of the knowledge obtained from a model that has already been trained on a large amount of data in a different domain [36]. The goal of TL is to leverage knowledge learned from a related task rather than training a model from scratch, which can not only reduce the amount of required training data but also accelerate the training of models for an ASD diagnosis. This approach has been highly successful, delivering top performance in benchmark tests and real-world applications. Models in computer vision often use pre-trained CNNs such as ResNet [37,38] and VGG [39], which are used as the backbone with the final layers adjusted to fit the ASD classification task [5,12,14,17,40]. Furthermore, TL can offer solutions to challenges in brain disorders research by facilitating the replication of findings using larger, more diverse datasets [12].

This research aims to investigate the utilization of recent advances in transfer learning for an ASD diagnosis, with a specific focus on the application of pre-trained recently developed vision transformers and a simple yet efficient 3D-CNN model. To the best of our knowledge, no previous studies have explored the use of TL with the “ConvNeXt for the 2020s” family of transformers dubbed as ConvNeXt [41], MobileNet [42], Swin [43], and ViT [44] in the context of ASD diagnosis using raw functional (fMRI) and structural (sMRI) modalities. While some recent studies initiated this direction by using VGG and ResNet for ASD diagnosis [5,12,14,17,40], there is room for research directed to applying vision transformers trained on rich data and fine-tuned them using raw sMRI and fMRI modalities with different brain views. Furthermore, this study not only emphasizes the use of MRI as image data for classification purposes but also more importantly explores multi-slice generation techniques from these MRI modalities. Additionally, we investigate the potential of directly utilizing raw 3D MRI data with 3D-CNNs for ASD diagnosis. Therefore, in this research work, we aim to contribute to the advancement of ASD diagnosis by exploring the synergies between transfer learning with vision transformers and the utilization of raw 3D MRI modalities with 3D-CNNs. The contributions of this work are highlighted in the following points:

The present study explores the effectiveness of multi-slice extraction mechanisms from raw MRI modalities as follows:

- Extracting 1, 10, and 50 slices along each brain plane (axial, sagittal, and coronal) to generate sequences of 2D images from raw 3D sMRI scans.
- Extracting 10, 30, and 50 slices along all brain planes (axial, sagittal, and coronal) to generate sequences of 3D images from raw 4D fMRI scans.
- Extracting all slices or giving some exceptions to the beginnings and the ends along all brain planes (axial, sagittal, and coronal) to generate sequences of 3D images from 3D sMRI and 4D fMRI scans.
- The study sheds light on the success of TL approaches by utilizing up-to-date vision transformer models for ASD diagnosis. ConvNeXt [41], MobileNet [42], Swin [43], and ViT [44] were used in conjunction with 2D sMRI-generated data.
- The study explores the diagnostic capability of the proposed 3D-CNN model to automatically leverage ASD biomarkers from 3D sMRI and fMRI generated data, and the classifiability of ASD subjects versus typical control (TC) subjects.
- Finally, this research contributes to the progress of the ASD field by evaluating the proposed methods with state-of-the-art models that have utilized either transfer learning or 3D-CNN models. These models are considered benchmarks for comparison with our models, as they also made use of the same dataset that we used in our study.

The rest of this paper is organized as follows. Section 2 discusses some of the relevant works that investigated deep learning and transfer learning models for ASD diagnosis pre-trained on neuroimaging data. Section 3 shows the proposed methodology used in this study to achieve the objectives of this work. In Section 4, the experimental setup, dataset exploration, resources, and tools are stated. In Section 5, the experimental results and statistical results are presented. Finally, in Section 6, concluding remarks and future directions are given at the end of this study.

## 2. Related Works

Different research works have utilized various MRI modalities with their respective analysis methods and with a particular focus on DL and TL techniques. Autoencoders were utilized in various ways for ASD detection [25,27,28,29]. For example, a study by Devika, Mahapatra, Subramanian, and Oruganti [29] utilized an autoencoder to enable low-dimensional embedding of high-dimensional sMRI input for ASD diagnosis. The study used a small dataset of 23 ASD and 15 TC collected from UCLA and UPSM sites in the ABIDE repository. From the 3D sMRI, 60 slices along the axial, coronal, and sagittal planes were used to train their model. The maximum accuracy of 86.95% was achieved by training the autoencoder exclusively on TC scans and considering ASD scans as outliers. Since many researchers used dimension reduction and normalization techniques from 4D/3D MRI to 2D images, many have employed CNNs to classify the subjects into ASD or TC subjects [2,12,13,14,15,16,17,18,19,20,21,22,23,24,25,26], To exemplify some studies, the DarkASDNet was proposed by Ahammed, Niu, Ahmed, Dong, Gao, and Chen [26] to perform ASD diagnosis on 3D fMRI collected from the NYU dataset. The model utilized the last 50 slices from the raw fMRI as input and trained using 20 convolutional layers and 6 maxpooling layers. Few studies used raw 3D MRI scans as inputs for the ASD classifier. One study by Shahamat and Abadeh [13] proposed 3D-CNN for ASD diagnosis utilizing 3D MRI ABIDE datasets. A genetic algorithm brain masking method was applied to discover brain regions with larger discrimination features. The model was trained with a cross-entropy loss function and Adadelta optimizer which achieved a classification accuracy of 70%.

Researchers have been able to improve accuracy and efficiency in ASD diagnosis through the application of TL and leveraging pre-existing model knowledge. TL models like VGG and MobileNet-V1 have achieved high classification accuracies of 90–95% when applied to facial images [45,46]. However, the utilization of TL using MRI data for ASD diagnosis remains unexplored which could be due to the complexity of 4D and 3D images in comparison with 2D images, and the computational burden caused by such sized data. We have found few studies primarily exploring TL using VGG and ResNet architectures [5,12,14,17,40]. An example of two studies is given here. A study by Tang, Kumar, Chen, and Shrivastava [40] proposed a ResNet network with 18-layer to extract meaningful representations from fMRI scans. The ABIDE dataset was utilized in its entirety. To process the 3D fMRI data, all 2D convolution layers in the ResNet were replaced with 3D convolution layers. The ResNet was trained from scratch for 30 epochs, resulting in a classification accuracy of 73%. Following this, Sharif and Khan [5] employed VGG for ASD diagnosis by modifying the last fully connected layer to perform ASD/TC classification using sMRI data from the ABIDE dataset. The VGG model was trained with a cropped portion of sMRI that includes the corpus callosum region in the subject’s brain. During training, the model utilized the softmax function and ADAM optimizer, achieving a classification accuracy of 66%. The remaining studies will be discussed shortly in the baselines section, as they serve as the benchmarks for comparison with our models, as they also utilized the same dataset that we used in our study.

## 3. Methods

### 3.1. General Framework of the Proposed Methods

In this section, we present the proposed framework for ASD diagnosis using sMRI and fMRI modalities data. This study focuses on analyzing and exploring various image generation techniques from a standard dataset to classify images into predefined ASD or typical control (TC) subjects. Because this work investigates the effectiveness of multi-slice generation, vision transformers, and 3D-CNNs, we divided our research framework into two parts: the first for the methods used with sMRI modalities, and the second for those implemented with fMRI modalities. 

**Figure 1:**
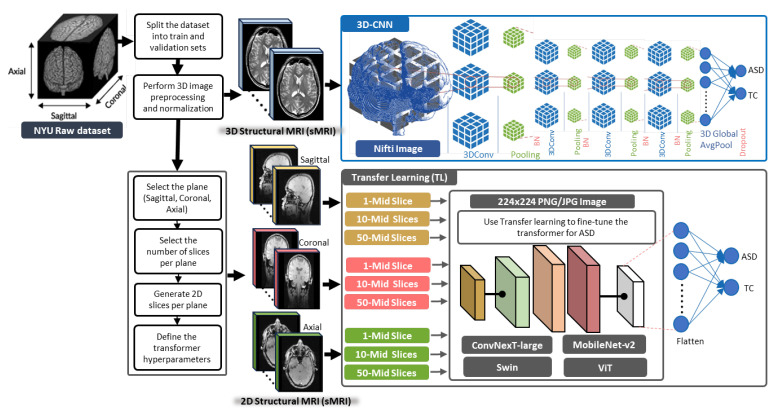
The proposed methodology for ASD classification using sMRI modalities, a 3D-CNN model, and TL with vision transformers.

Figure 1 shows the proposed methodology for ASD classification using sMRI modalities. To avoid data leakages between training and testing examples [47], the raw MRI dataset was first split into 80% training and 20% validation sets based on subjects before applying any further data generation. Then, to prepare the sMRI modalities, the raw 3D data was preprocessed and normalized using Fuzzy C-means (FCM)-based tissue-based mean normalization [48]. In FCM-based tissue-based mean normalization, the algorithm is applied to cluster the image pixels into different tissue groups based on their intensity values. The mean intensity value of each tissue cluster is then calculated. These mean values serve as reference points for normalization. By normalizing the intensities of the image pixels to the respective tissue mean values, the FCM-based tissue-based mean normalization ensures that the image intensities are adjusted to a consistent scale. This normalization step can be beneficial for ASD image analysis and classification. The resulting 3D sMRI images were used as input to 3D-CNN to perform ASD diagnosis. The proposed 3D-CNN architecture employed four sequential 3D convolutional layers (3DConv) followed by max pooling and batch normalization (BN). This approach aimed to leverage the spatial information present in the 3D sMRI data for accurate diagnosis. To implement TL, 2D sMRI images were generated from the 3D sMRI data by slicing along various anatomical planes namely axial, coronal, and sagittal. The number of extracted slices for each plane was determined by choosing 1, 10, and 50-middle slices per plane. We leveraged the constructed slice representations as input for four vision transformers and trained these models separately on each plane. The performance of each model was evaluated based on their loss, accuracy, and F1 score. In the subsequent sections, more details about TL and 3D-CNN are given.

**Figure 2:**
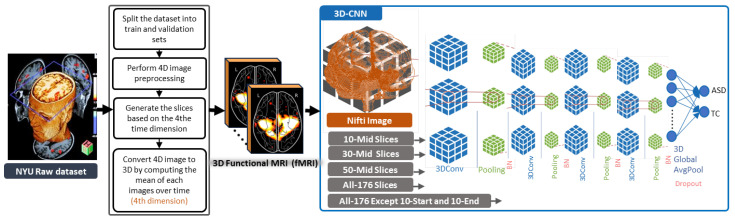
The proposed methodology for ASD classification using fMRI modalities using 3D-CNN models.

As illustrated in Figure 2. the fMRI modalities are processed differently. As fMRI are 4D images representing simple time-series or multi-volume data, different numbers of slices were generated based on the fourth dimension (i.e., time) whereby the first three dimensions represent axial, sagittal, and coronal planes. Specifically, 10, 30, 50, all slices, and all slices except 10-start slices and 10-end slices were considered. To calculate the mean of the images over time or the fourth dimension, it is important to note that if a list of 4D images is provided, the mean of each individual 4D fMRI image is computed separately, and then the resulting means are computed together resulting in 3D fMRI images. These 3D images were then normalized FCM-based tissue-based normalization [48], and used as input to the proposed 3D-CNN, following a similar architecture as the sMRI experiment. The objective of using the same 3D-CNN model was to assess the performance of ASD diagnosis using the generated sMRI and fMRI datasets.

### 3.2. 3D-CNN Architecture for ASD Diagnosis

This study proposed a 3D-CNN model architecture that is simple yet effective in analyzing 3D neuroimaging data. The model uses raw 3D images from sMRI and fMRI neuroimages collected from the NYU dataset. Before training the model, the neuroimages are preprocessed to ensure consistent data representation through normalization and resizing. This involved normalization and resizing steps to enhance comparability and standardize spatial dimensions. The CNN architecture involved an input layer, followed by a 3D convolutional layer with 64 filters of size 3 × 3 × 3 and a ReLU activation function. Next, a max pooling layer with a pool size of 2 × 2 × 2 was used. Batch normalization (BN) was applied after each convolutional and max pooling layer to enhance the stability and efficiency of the model. The layers were repeated for an additional three convolutional layers, each with progressively more filters: 64, 128, and 256. After the convolutions, a global average pooling layer was applied to obtain a global representation of the extracted features. Next, a dense layer with 512 units and ReLU activation was employed to flatten the extracted feature weights. To prevent overfitting, a dropout regularization rate of 0.3 was applied to the dense layer. Finally, the output classification layer was composed of a single unit and a sigmoid activation function for binary classification, distinguishing between ASD and TC subjects. The model was trained for 50 epochs, using a binary cross-entropy loss function, the Adam optimizer with a learning rate of 0.001, and evaluated using loss, accuracy, and F1 metrics.

### 3.3. TL Vision Transformers for ASD Diagnosis

Following the success of transformers in NLP, many researchers have begun to explore transformer architectures for computer vision tasks [41,43,44,49,50], and very recently for medical image analysis [51,52,53]. CNNs and their variants provide state-of-the-art performance, partially due to their expanding receptive fields that lead to learning hierarchies of structured image representations. Generally, the idea of capturing visual meaning in pictures is considered the foundation of successful computer vision networks. However, conventional CNNs have the limitation of ignoring long-term relationships between objects in the image [52]. Studies have shown that adding the attention mechanism, which has been successful in NLP, to CNNs can help capture these long-term dependencies and improve image classification accuracy by treating each image as a sequence of patches [43]. In this context, this study employed four vision transformers, namely ConvNeXt [41], MobileNet [42], Swin [43], and ViT [44] in the context of ASD diagnosis using sMRI modalities. 

The ConvNeXt transformers family developed by Liu, Mao, Wu, Feichtenhofer, Darrell, and Xie [41] was specifically designed for image classification tasks. It incorporates elements from the ResNet architecture and has undergone pre-training on a large dataset of images. This pre-trained ConvNeXt model serves as a robust foundation, as it has acquired the ability to extract meaningful features from diverse image data. The versatility of the ConvNeXt transformer extends beyond image classification, making it applicable to a wide range of image-related tasks, including object detection, image segmentation, and recognition. The pre-trained models offer customization options with varying layer sizes, input sizes, and training datasets. Notably, ConvNeXt benefits from residual connections, enhancing accuracy by efficient information propagation throughout the network. As there is a family of ConvNeXt transformers, we chose one of them named ConvNeXtXLarge for ASD classification.

The MobileNet transformer was designed by Sandler, Howard, Zhu, Zhmoginov, and Chen [42] for mobile devices and low-power applications. It incorporates depth-wise separable convolutions, which involve a depth-wise convolution followed by a point-wise convolution. This design choice significantly reduces the number of parameters in the network, resulting in lower computational requirements for both training and inference. The MobileNet was pre-trained on large-scale datasets like ImageNet and is commonly utilized in computer vision tasks such as image classification, object detection, face recognition, and scene understanding. The MobileNetV2 is the variant that we used in this study.

The Swin transformer was proposed by Liu, Lin, Cao, Hu, Wei, Zhang, Lin, and Guo [43] as a variant of the vision transformer architecture that utilizes a shifted window algorithm for classification. It achieves hierarchical representation by starting with small-sized patches and gradually merging neighboring patches while decreasing the amount of computation needed to calculate the attention of high-resolution images. Swin was pre-trained on ImageNet-1K, ImageNet-22K, COCO 2017, and ADE20K, and has shown remarkable performance in image classification, object detection, and semantic segmentation. As there are multiple variants of Swin, this work employed the SwinV2Tiny256.

The ViT is an image classification transformer developed by Dosovitskiy, Beyer, Kolesnikov, Weissenborn, Zhai, Unterthiner, Dehghani, Minderer, Heigold, Gelly, Uszkoreit and Houlsby [44] which provides new insight for vision-related tasks, which are markedly distinct from the current state-of-the-art approaches based on CNNs. Although the original transformer model combines both encoders and decoders, the ViT model only has encoders pre-trained on a vast collection of images in supervised learning, including ImageNet-1K, ImageNet-21k, and JFT datasets. This work employed the ViT_base16 model.

Furthermore, in this study, we focused on the downstream task of ASD diagnosis using these vision transformers and utilized pre-built modules offered by the Keras library. To accomplish this objective, we collected 3D sMRI neuroimages from the NYU dataset in the ABIDE-I repository. Before proceeding with the data generation process, we divided the subjects’ images into train and test sets, allocating 80% for training and 20% for testing. The vision transformers employed in this study required 2D images as input with 3 channels. Thus, we followed a specific process for multi-slice generation as we outlined in the experimental setup section to prepare the 2D sMRI data. After generating the 2D images, we performed normalization and resized them to a resolution of 224 × 224 pixels. In cases where the images were grayscale, we duplicated the image to create three input channels. In order to further enhance the diversity and size of our dataset, we applied a data augmentation pipeline which consists of two augmentation layers: random flip and random rotation. By employing these data augmentation techniques, our aim was to increase the size and diversity of our dataset. The visualization of augmented data will be presented in the experimental setup section. 

Our approach for utilizing the vision transformers involved excluding the top layer from the original architecture and adding a classification layer. During the training, the TL model architectures were trained using identical hyperparameter settings to maintain consistency. The training process was configured to run for 50 epochs, utilizing a binary cross-entropy loss function and the Adam optimizer with a learning rate of 0.001. Upon completion of the training and evaluation process, we reported the classification loss, accuracy, and F1 score for all transformers.

## 4. Experimental Setup

### 4.1. Datasets

**Figure 3:**
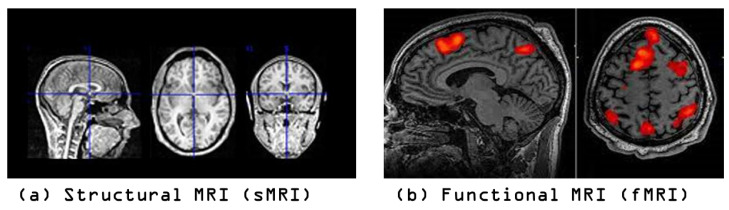
Structural MRI (sMRI) is a non-invasive technique for examining the anatomy and pathology of the brain (**a**), as opposed to using functional MRI (fMRI) which examines the brain’s activity (**b**) (photos by The University of Edinburgh) (https://www.ed.ac.uk/clinical-sciences/edinburgh-imaging/research/themes-and-topics/medical-physics/imaging-techniques, accessed on 20 March 2023).

MRI is a non-invasive brain imaging technique that enables the quantification of biomarkers for accurate diagnosis of neurological disorders like schizophrenia, ASD, and Alzheimer’s diseases [54]. It can be classified into structural and functional scans based on the scanning techniques used. The sMRI scans analyze irregular neuroanatomy using volumetric and morphometric investigations based on the three acquisition planes of axial, coronal, and sagittal [29]. They are widely used in clinical studies due to their ability to identify precise changes in brain structure and provide images with higher contrast and spatial resolution [29]. In contrast, fMRI scans can examine the brain’s activity by measuring blood flow to different parts of the brain. The brain is represented in fMRI data as a collection of voxels, with each voxel’s activity measured over time and represented as time series data [23,28]. Figure 3 shows an example of both modalities of the sMRI and fMRI. 

This study utilized the ABIDE-I repository, which is a well-known publicly available dataset in the field of ASD research (For additional information on ABIDE I, please visit http://fcon_1000.projects.nitrc.org/indi/abide/, accessed on 20 March 2023). The primary purpose of ABIDE-I was to explore the neurological underpinnings biomarkers of ASD, and it comprises sMRI, corresponding fMRI, and information about the characteristics of both individuals with ASD and TC subjects. ABIDE-I was collected from 17 distinct sites around the world, each with unique characteristics regarding subjects and imaging protocols, resulting in a highly complex and heterogeneous mix of subjects and imaging modalities. In this study, raw 3D sMRI and 4D fMRI neuroimaging datasets from the NYU site were used from the ABIDE repository. We meticulously selected our dataset from the NTU subset for several reasons:

- The ABIDE dataset includes a heterogeneous mix of subjects and imaging modalities with a variety of scanning methods, and some datasets of lower quality, which could impact the outcomes of our study.
- Numerous research articles have highlighted the NYU site (such as [12,17,26]), citing their superior scanning techniques and the thorough preparation of data for both typical control (TC) and ASD individuals.
- Our decision to use data exclusively from a single site enhanced our findings, and we plan to scale our future research to include additional sites that maintain the same level of scanning and data quality as NYU.

Table 1 summarizes the phenotypic characteristics of the subjects in the dataset. The sMRI was collected directly from the NYU site which consists of 79 ASD and 105 TC sMRI scans. The preprocessed version of fMRI was downloaded through the Configurable Pipeline for the Analysis of Connectomes (CPAC). The processing procedures for the fMRI data include skull stripping, motion correction, slice timing correction, normalization, motion realignment, band-pass filtering of 0.01–0.1 Hz, and registration of fMRI images into the standard anatomical space. During the preprocessing stage, some subjects were excluded from the study due to low image quality or missing scans. For this study, a total of 74 ASD and 98 TC fMRI images from the NYU site were included.

### 4.2. Slice Extraction from sMRI Modalities

**Figure 4:**
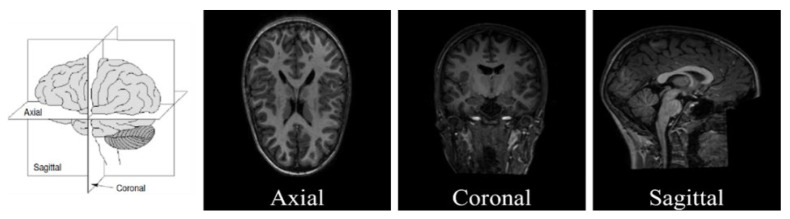
Axial, sagittal, and coronal planes used in sMRI modalities.

**Figure 5:**
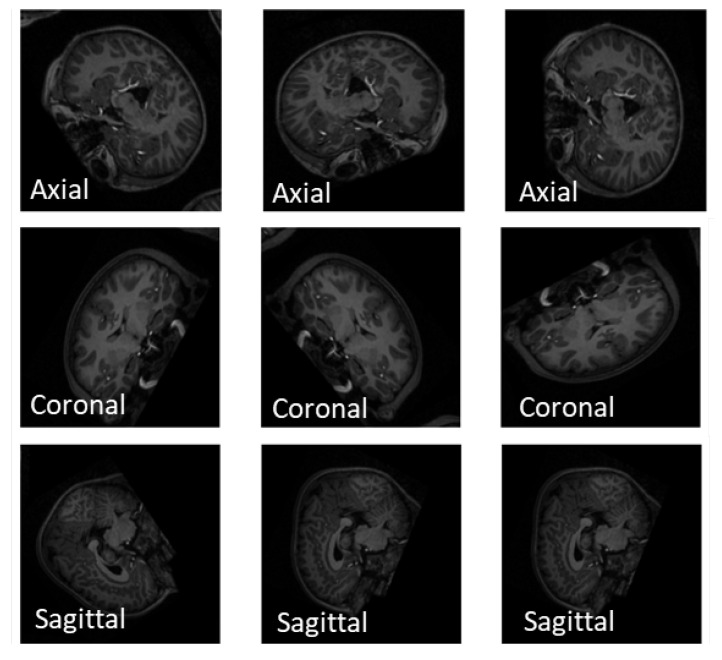
Augmentation techniques of sMRI data along the axial, sagittal, and coronal planes.

Recent vision transformers are designed to handle 2D images, necessitating the preprocessing of raw 3D sMRI data to ensure their compatibility with the transformers. To convert the 3D sMRI images into 2D, we can extract 2D slices along the three main brain planes: the axial, coronal, and sagittal planes. Visualization for the three planes can be seen in Figure 4. In the 3D coordinate system, the brain can be divided into three planes, from front to back: Sagittal, side to side: Coronal, and top to bottom: Axial. The sMRI images have a format of NIfTI (.nii) which is a common file format used to store medical imaging data. The images were first loaded using the nibabel library which provides tools for reading, writing, and manipulating neuroimaging data. Typical sMRI scan usually has 128–256 slices along each plane, and the middle slice sequence is known to contain a majority of the vital brain information as claimed by Devika, Mahapatra, Subramanian, and Oruganti [29]. In this study, we utilized three primary methods to generate multi-slice data. The total number of the generated image datasets is summarized in Table 2. Before applying any data generation methods, we divided the subjects into an 80% training set and a 20% testing set which preservers our experiments from data leakages between training and testing examples [47]. The first method involved extracting a single representative slice from the middle of each subject. The three brain planes, namely the axial, coronal, and sagittal planes, correspond to the z-axis, y-axis, and x-axis, respectively. To obtain the middle slice of the axial plane, we accessed the image at coordinates (x, y, z/2), where z represents the number of slices along the z-axis. We repeated this procedure to extract the middle slices along the coronal and sagittal planes. Once the middle slices were extracted, we scaled them to a size of 224 × 224 pixels and saved them in PNG format. These 2D slice images were then organized into three datasets, one for each brain plane. We also explored alternative data generation methods by extracting 10 and 50-middle slices along each of the three planes. For this, we selected a specific plane from the 3D sMRI data and retrieved the middle slices by specifying the number of slices to be extracted. The resulting slices were resized to a resolution of 224 × 224 and saved as PNG images. Additionally, data augmentation was implemented on the slices generated from all planes before training our proposed models. Figure 5 exemplifies the results from simple augmentation methods including random flip and random rotation using the slices generated from each of the axial, sagittal, and coronal brain planes.

### 4.3. Slice Extraction from fMRI Modalities

**Figure 6:**
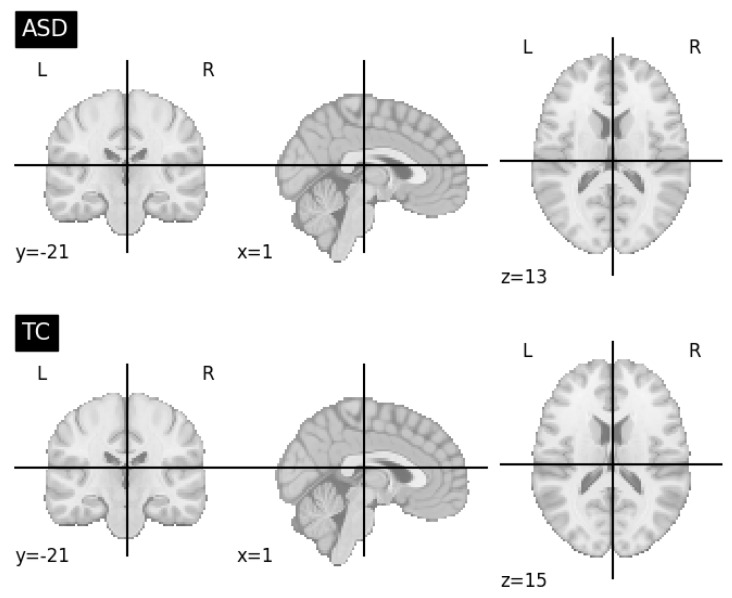
Visualization of fMRI modalities along the axial, sagittal, and coronal planes using the nilearn.image.mean_img to generate a single 3D image from the 4D fMRI data.

Prior to using the fMRI data as input for our models, we performed several data preparation steps. Our objective was to obtain 3D fMRI images that effectively captured the functional brain activity related to ASD. To obtain the 3D fMRI images, we explored different slice retrieval strategies. Initially, we retrieved the whole slices from the 4th dimension of the fMRI data for each subject. To elaborate, we investigated the use of the entire set of 176 slices for each subject. This approach allowed us to utilize the complete temporal dimension of the fMRI data, providing a comprehensive representation of the functional brain activity. These retrieved slices were then averaged to generate a single 3D fMRI image. Additionally, we applied a normalization step to ensure consistency and comparability between the volumes in the fMRI image. By normalizing the fMRI data, we aimed to reduce potential variations caused by differences in acquisition parameters or intensity scaling. Figure 6 shows a visualization of fMRI modalities along the axial, sagittal, and coronal planes using the nilearn.image.mean_img to generate a single 3D image from the 4D fMRI data. 

**Figure 7:**
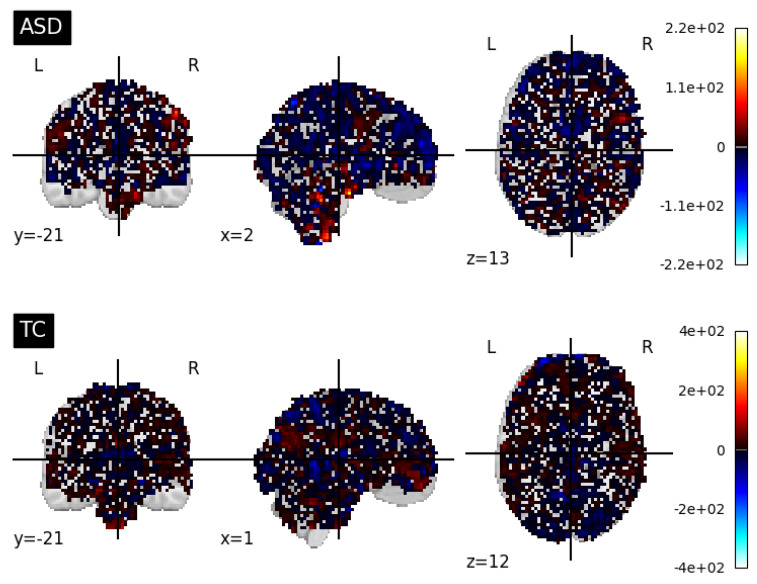
Visualization of extracted 30-middle slices fMRI modalities along the axial, sagittal, and coronal planes using the image.index_img(subject, slice(73, 103)) to extract slices and the nilearn.image.mean_img to generate a single 3D image from the 4D fMRI data.

In addition to the retrieval of the whole slices of image data, we also explored alternative slice retrieval strategies. We experimented with retrieving 10, 30, and 50-middle slices from the 4th dimension of the fMRI data. These retrieved slices were processed in a similar manner, involving the calculation of the mean average and normalization. Figure 7 shows samples of ASD and TC subjects of extracted 30-middle slices fMRI modalities along the axial, sagittal, and coronal planes using the image.index_img(subject, slice (73, 103)) to extract slices, and the nilearn.image.mean_img to generate a single 3D image from the 4D fMRI modalities. To further explore the influence of slice retrieval, we also considered a variation of using the whole 176 slices except 10 slices from the beginning and the end. In this case, we excluded the first and last 10 slices from the 176 slices, aiming to minimize potential artifacts resulting from the initial and final phases of the fMRI scan. We believed that the exception of the slices from the beginnings and the ends may reduce irrelevant (mostly black) slices, keeping the most informative ones. By varying the number of retrieved slices, we aimed to capture different levels of temporal information and evaluate their impact on the subsequent ASD diagnosis. These steps ensured that the fMRI images effectively represented the functional brain activity related to ASD, enabling the CNN model to extract relevant features for accurate diagnosis. 

### 4.4. Baselines

Using MRI modalities, three recent studies used VGG and ResNet for ASD diagnosis, MCSE-VGG [17], Deep Ensemble Learning—VGG16 [12], and CNN—ResNet [14]. Thus, our proposed methods were compared with these baselines to benchmark our work and provide linkage with the literature studies. The first baseline [17] was originally designed using a multi-channel squeeze and excitation VGG architecture. The squeeze and excitation modules were utilized to enhance the model’s performance by considering the interdependencies between channels. The VGG-net was utilized for ASD diagnosis classification, with the 3D convolution replaced with the 2D convolution. During the model’s training phase, the ADAM optimizer was utilized with an initial learning rate of 0.0005. If the validation loss did not decrease after 10 epochs, the learning rate was subject to decay of 0.1. The model was trained using a batch size of 12, and a LeakyReLU activation with a negative slope of 0.01 was employed. Convolution kernel parameters were regularized using L2 norms. The cross-entropy loss function was used to determine whether a subject belonged to the TC or ASD class. The second baseline [12] employed the VGG16 model to extract relevant features from the glass brain and stat map images generated from fMRI. These extracted images were then fed into an ensemble learning classifier for ASD diagnosis. A total of six convolutional layers, four max-pooling layers, two batch normalization layers, one flattens and dropout layer, and two densely connected layers followed by a sigmoid activation function were used in their network. A class-wise cross-entropy was used for the loss function, and L1 regularization was applied to penalize the sum of the absolute weights. Ensemble learning frameworks by adopting the VGG16 model were explored as feature extraction, and the feature maps of a convolutional base were the presence maps of generic notions that were more reusable. In each ensemble classifier, predictions for each pipeline were averaged based on the two types of images, and the final evaluation metrics were calculated as the arithmetic mean of the specific binary class predictions. Using the ADAM optimizer, each ensemble model was trained for 1000 iterations with a learning rate of 0.0004, with the final layer activation set to the sigmoid function. In the third baseline [14], morphological features were extracted from sMRI and used as input to: the CNN classifier and pre-trained ResNet. In CNN classifiers, convolution and pooling operations were used to extract relevant features. Batch normalization was adopted after each convolution and before activation. Following these layers, fully connected layers performed classification on the output. ResNet was used to learn both local and global features via skip connections combining different levels, addressing the issue that plain networks were unable to integrate different levels. They trained ResNet from scratch and initialized the weights randomly. During the training, they utilized Adam optimizer, batch size of 32, and learning rate of 0.00001. Results from these benchmark studies and the present one will be discussed shortly in the results section.

### 4.5. Evaluation

In this study, we used accuracy, F1, and loss functions to evaluate the proposed models for ASD diagnosis using sMRI and fMRI modalities. We divided the dataset into training (80%) and testing (20%) sets. Then, we trained the models using the training data and assessed how well the models performed using the testing set. Using Equation (1), the models’ accuracy was calculated based on the ratio of correct predictions to total predictions, with N representing the number of subjects in the test set and C representing the number of correctly classified subjects. This measure provides an evaluation of the model’s performance in detecting ASD subjects, with high accuracy suggesting that the model is successful in classifying the data. The F1 measure was computed using Equation (2), which is the harmonic mean of both precision and recall. In this measure, precision and recall were combined into a single metric, where the product was divided by the average. The cross-entropy loss function was used to evaluate the model’s ability to generalize to unseen data in the testing set. The cross-entropy loss was computed using Equation (3), where $q_{i}\left(x\right)$ represents the predicted probability of the output belonging to the i-th class, and $p_{i}\left(x\right)$ represents the true probability [36]. These measures were chosen for their effectiveness in evaluating the classification model performance, particularly in the context of neuroimaging research.

(1) Accuracy=C/N

(2) F1−Score=2×(Precision×Recall)/(Precision+Recall)

(3) $$H\left(X\right)=-\sum_{i=1}^{n}p_{i}\left(x\right)\log(q_{i}(x))$$

### 4.6. Resources and Tools

The main resource utilized in this research for executing the transformers is Google Colab Pro+. It is a cloud-based computing platform that gives access to high-performance computing resources such as GPUs and TPUs to help with deep learning model building and deployment. We chose the GPU A100 runtime in Google Colab Pro+ which includes an NVIDIA A100 GPU with 83 GB of system RAM, 40 GB of GPU RAM, 500 processing units, and 166 GB of disk space. This resource allowed us to formulate and train the transformers quickly and effectively, resulting in high levels of accuracy in our classification performance. The training time for the transformers ranged from 30 min to 5 h depending on the size of the data. We implemented model training and testing using the Keras library, which offers a variety of tools and modules for constructing and training the models. The MRI modalities were preprocessed with Nilearn, Nibabel, PIL, and NumPy libraries. Nilearn and Nibabel are two open-source Python libraries that are widely used in neuroimaging research for data visualization, analysis, and modification. These two libraries, when combined, provide a robust and versatile collection of tools for manipulating neuroimaging data. For 3D image normalization, FCM-based tissue-based normalization was used from an intensity-normalization package specifically designed for medical images [48].

## 5. Experimental Results

### 5.1. ASD Classification Results from sMRI Modalities 

In this study, we conducted two experimental sets using sMRI for ASD diagnosis and classification into TC or ASD. The first experimental set involved generating 2D slices along three brain planes (Axial, Coronal, and Sagittal) from the sMRI images. We explored different numbers of generated slices, namely 1, 10, and 50 slices, to examine their impact on the diagnostic performance. Data augmentation techniques were applied to augment the dataset, and four vision transformers (ConvNeXt [41], MobileNet [42], Swin [43], and ViT [44]) were trained separately on each dataset. All the transformers were trained with the same hyperparameter settings. We evaluated the performance of each transformer using accuracy, loss, and F1 score as metrics. The experimental results are presented in Table 3 for each dataset, where the maximum performance in terms of accuracy and F1 score are highlighted in bold. To elaborate on the results obtained by the sMRI modalities, initially when one slice per subject was extracted along the three planes, the accuracies ranged from 58% to 77%. Among the transformers, the ConvNeXt achieved the highest accuracy of 77% and an F1 score of 76% along the Axial plane. For the Coronal and Sagittal planes, the ConvNeXt also outperformed the other transformers with accuracies of 69%, suggesting its effectiveness in capturing relevant features from a single slice. Next, in the 10-slices experiment, the dataset was expanded by extracting more slices to represent each subject. The accuracy ranged from 58% to 71% for all transformers. The maximum accuracy of 71% and an F1 score of 68% were achieved with the ConvNeXt along the Axial plane. Along with the Coronal and Sagittal planes, the MobileNet achieved the highest accuracy of 69%, while the ConvNeXt achieved an accuracy of 63%. For the 50-slices experiment, our aim was to capture more structures of the subjects’ brains by including 50-middle slices, which contain the majority of the brain structure and volumetric data. However, the accuracies ranged from 58% to 68% without significant improvements. Again, the ConvNeXt achieved accuracies of 65%, 68%, and 66% along the Axial, Coronal, and Sagittal planes, respectively. This indicates that simply increasing the number of slices does not necessarily lead to better diagnostic performance and that the ASD biomarkers may not be clearly seen by the models using the sMRI modalities. 

The second experimental set involved using the raw 3D sMRI images as input and employed 3D-CNN architecture to fully capture the brain structure of the subjects in our experiments. The 3D-CNN was trained by utilizing all subject slices. The achieved results were a loss of 72%, accuracy of 65%, and F1 score of 57%. This suggests that the 3D-CNN while providing a holistic approach by considering the entire volume of the brain, achieved comparable results to the vision transformers using the sMRI modalities.

### 5.2. ASD Classification Results from fMRI Modalities

**Figure 8:**
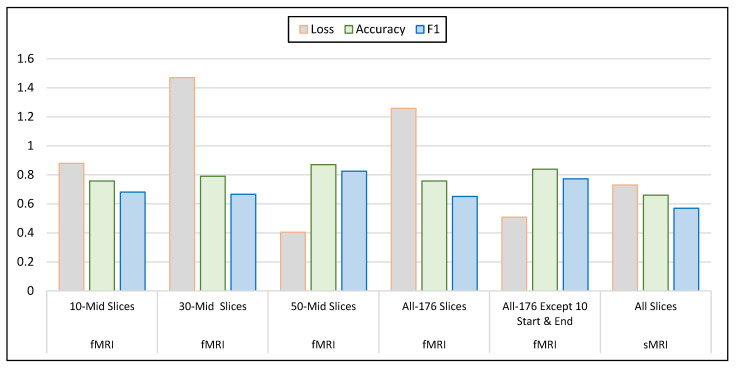
Comparison of 3D-CNN performance metrics using sMRI and fMRI 3D modalities.

In another part of our experimental work, we utilized fMRI images as input for the 3D-CNN models and evaluated their effectiveness for ASD classification. A summary of the results for the CNN experiments, using different slice retrieval strategies, is reported in Table 4 where the maximum performance in terms of accuracy and F1 score are highlighted in bold. As can be seen in the table, we observed that the 3D-CNN model with fMRI models achieved state-of-the-art results where the accuracy ranged from 75% to 87% across different slice retrieval strategies. The maximum accuracy and F1 score achieved by the 3D-CNN model was 87% when using 50-middle slices from the fMRI images. This confirmed the previously reported literature that the 50-middle slices contain a significant amount of information about the functional brain activity related to ASD. Furthermore, the retrieval of all slices except the 10 start-end slices also achieved promising results, with an accuracy of 83% and an F1 score of 77%. This indicates that excluding the initial and final phases of the fMRI scan, which may be more prone to artifacts, did not significantly impact the diagnostic performance. The achieved accuracies demonstrate the effectiveness of the 3D-CNN architecture in extracting relevant features from the fMRI data. As can be seen in Figure 8, a comparison of 3D-CNN performance metrics using sMRI and fMRI 3D modalities along the axial, sagittal, and coronal planes reported that using 50-middle slices of fMRI images achieved the highest accuracy and F1 scores and the lowest loss metrics and such results can be considered state-of-the-art for ASD classifiers of ASD versus TC subjects.

### 5.3. Comparison with the Baselines for ASD Diagnosis

For each MRI type, we conducted separate comparisons to evaluate the performance of our proposed approach for ASD diagnosis. Our results from sMRI modalities were compared to those of an ASD diagnosis study that used sMRI and a pre-trained model [14]. Furthermore, our results from fMRI modalities were compared to baseline studies that used fMRI with a pre-trained model to diagnose ASD [12,17]. A comparison between the results obtained by the proposed method and the benchmarks is summarized in Table 5 (for fMRI) and Table 6 (for sMRI).

**Figure 9:**
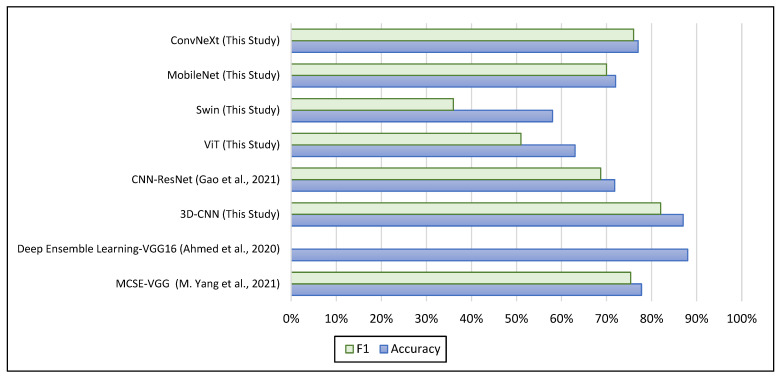
Comparison of the performance metrics using the proposed approaches and the benchmarks [12,14,17].

As can be seen in Table 5, the study by Yang, Cao, Chen, Chen, Fan, Li, Wang, and Liu [17] represented each subject by their brain functional network, and these data were used as input to train the MCSE-VGG. The VGG model was used as a backbone for training their model, and they achieved a maximum accuracy of 77.74%. Their approach focused on leveraging the functional connectivity patterns within the brain network to identify ASD-related features. In the work by Ahmed, Zhang, Liu, and Liao [12], they used the VGG model as a feature extractor to extract features from glass brain images generated from fMRI. Large numbers of the generated images were used for the training, with 32,200 images generated using the NYU dataset. Remarkably, their approach achieved an accuracy of 88%. In comparison, our experimentation with the NYU dataset involved utilizing the mean of the 50-middle slices from the raw fMRI data which achieved a comparable accuracy of 87%. While our approach differs in terms of the specific methodology, it is noteworthy that our accuracy is comparable to the state-of-the-art results in ASD classification. Another benchmarking presented in Table 6, showed that the pre-trained ResNet model was used as a part of their CNN classifier to perform the ASD diagnosis and they achieved maximum accuracy of 71.81% [14]. Their work extracted morphological covariance brain networks from sMRI as input for their model. Unlike our approach, this baseline study extracted features from sMRI rather than using sMRI as image data. In contrast, our study took a different approach by extracting 2D slice images from sMRI and training transformer models on the NYU dataset. The maximum accuracies achieved by our transformer models were 63%, 58%, 72%, and 77% for ViT, Swin, MobileNet, and ConvNeXt, respectively. By directly utilizing the image data instead of relying on feature extraction, our models exhibited promising classifier accuracy for ASD diagnosis. Figure 9 highlights the final thoughts of the achieved results by our proposed approaches and the benchmark studies. Furthermore, our findings, as set against the benchmarks shown in Table 5 and Table 6, confirm that our results do not exceed those baselines. This acknowledges the reality that research efforts, including our own, which utilize raw data along with central slices from high-quality MRI modalities and apply transfer learning from pre-trained models, are capable of yielding comparable results.

## 6. Conclusions and Future Works

People with Autism Spectrum Disorder (ASD) struggle with communication, social interaction, and repetitive actions. Clinicians use various techniques, including MRI scans, to diagnose ASD. These scans provide important information about the structure and function of the brain. However, the traditional ASD diagnosis process using MRI scans is time-consuming and requires significant effort. In this study, we introduced a new approach for diagnosing ASD using MRI scans. We utilized four vision transformers and a 3D-CNNs model to assist in the diagnosis process. To evaluate the effectiveness of our approach, we used the NYU dataset from the ABIDE repository, which is a commonly used dataset in ASD research. We also employed different methods to extract 2D representations from the raw sMRI scans and different 3D slice retrieval strategies from raw fMRI modalities. Our results show that we achieved a maximum accuracy of 77% using sMRI data and a maximum accuracy of 87% using fMRI data. We also investigate the impact of the number of slices and the orientation of brain planes on the performance of our approach. We find that including more slices in the sMRI experiments does not significantly improve diagnostic performance. In the fMRI experiments, different slice retrieval strategies show variations in performance, highlighting the importance of selecting the right strategy to capture temporal information. Our use of the 3D-CNN architecture, which considers the entire brain volume, provides valuable insights for ASD diagnosis. Additionally, we compare our approach with the baseline benchmarking studies that use pre-trained models for ASD diagnosis and find that our models achieve higher or comparable results. 

In addition to the promising findings of this study, there are several potential areas for future research in ASD diagnosis. One avenue is the integration of multiple modalities, such as combining sMRI and fMRI data, which has the potential to enhance diagnostic accuracy by providing complementary information. Another promising direction is the utilization of pre-trained brain transformer models that have been trained on large and diverse neuroimaging datasets. These models have the potential to further improve ASD diagnosis accuracy. However, existing pre-trained brain transformers are currently limited in their applicability for ASD diagnosis, as they typically require training from scratch and lack available weights. Future research should focus on developing neuroimaging-specific pre-trained brain transformers with readily available weights for manual fine-tuning. Additionally, it is crucial to modify existing vision transformers to handle 3D input, as MRI data is typically acquired in 3D volumes, which offer more comprehensive information compared to 2D slices. Exploring methods for model interpretability, such as attention maps or saliency analysis, would provide valuable insights into the specific regions and features that drive diagnostic decisions. Collecting larger and more diverse datasets would also be beneficial in order to improve the generalizability of the models and capture a wider range of data heterogeneity [55]. We acknowledge that extending our subsequent investigations to encompass a larger dataset and introducing a third split (i.e., train, validate, and test) could greatly benefit the advancement of the field. While the current study utilized the NYU dataset, further validation on diverse and independent datasets from different populations and settings is necessary due to the limited availability of datasets for the development and evaluation of ASD diagnosis models. By pursuing these future directions, we can potentially assist clinicians in the early diagnosis and treatment of ASD, ultimately benefiting individuals with autism and their families.

## Figures and Tables

**Table 1:** Phenotypic information of subjects with autism spectrum disorder (ASD) and typical controls (TC).

Site	ASD			TC		
	AGE AVG	GENDER	TOTAL COUNT	AGE AVG	GENDER	TOTAL COUNT
NYU (sMRI)	14.51	11 F, 68 M	79	15.8	26 F, 79 M	105
NYU (fMRI)	14.76	10 F, 64 M	74	15.75	26 F, 72 M	98

**Table 2:** The number of slices (i.e., 2D images) generated from sMRI using multi-slice generation.

Site	3D sMRI	Brain View	No Slices/Plane	Train Set (80%) Case No		Test Set (20%) Case No	
				0952 to 1018	1036 to 1122	1019 to 1035	1123 to 1159
				ASD	TC	ASD	TC
NYU	ASD 79, TC 105	Axial	1	64	84	15	21
NYU	ASD 79, TC 105	Coronal	1	64	84	15	21
NYU	ASD 79, TC 105	Sagittal	1	64	84	15	21
NYU	ASD 79, TC 105	Axial	10	640	840	150	210
NYU	ASD 79, TC 105	Coronal	10	640	840	150	210
NYU	ASD 79, TC 105	Sagittal	10	640	840	150	210
NYU	ASD 79, TC 105	Axial	50	3200	4200	750	1050
NYU	ASD 79, TC 105	Coronal	50	3200	4200	750	1050
NYU	ASD 79, TC 105	Sagittal	50	3200	4200	750	1050

**Table 3:** Experimental results from the sMRI modalities along different plane views using multi-slice generation, TL vision transformers, and 3D-CNN.

Method	Brain Plane	No Slices	Loss	Acc	F1	No Slices	Loss	Acc	F1	No Slices	Loss	Acc	F1
ViT	Axial	1 slice	0.68	0.58	0.36	10 slices	0.69	0.63	0.51	50 slices	0.67	0.61	0.54
	Coronal	1 slice	0.68	0.58	0.36	10 slices	0.69	0.61	0.44	50 slices	0.68	0.59	0.43
	Sagittal	1 slice	0.67	0.58	0.36	10 slices	0.68	0.60	0.46	50 slices	0.68	0.58	0.36
Swin	Axial	1 slice	0.67	0.58	0.36	10 slices	0.67	0.58	0.36	50 slices	0.67	0.58	0.36
	Coronal	1 slice	0.67	0.58	0.36	10 slices	0.68	0.58	0.36	50 slices	0.67	0.58	0.36
	Sagittal	1 slice	0.67	0.58	0.36	10 slices	0.67	0.58	0.36	50 slices	0.67	0.58	0.36
MobileNet	Axial	1 slice	0.70	0.72	0.70	10 slices	0.67	0.68	0.62	50 slices	0.66	0.64	0.59
	Coronal	1 slice	0.71	0.66	0.58	10 slices	0.67	0.69	0.64	50 slices	0.65	0.65	0.59
	Sagittal	1 slice	0.67	0.61	0.59	10 slices	0.68	0.60	0.45	50 slices	0.66	0.64	0.58
ConvNeXt	Axial	1 slice	0.63	0.77	0.76	10 slices	0.64	0.71	0.68	50 slices	0.67	0.65	0.58
	Coronal	1 slice	0.64	0.69	0.66	10 slices	0.64	0.67	0.62	50 slices	0.64	0.68	0.61
	Sagittal	1 slice	0.66	0.69	0.68	10 slices	0.76	0.63	0.53	50 slices	0.68	0.66	0.61
3D-CNN	3D (Axial, Coronal, Sagittal)									All slices	0.73	0.66	0.57

**Table 4:** Experimental results from fMRI modalities using multi-slice generation and 3D-CNN.

Method	Brain Plane	No Slices	Loss	Accuracy	F1
3D-CNN	3D (Axial, Coronal, Sagittal)	10-Mid Slices	0.8793	0.7581	0.6809
	3D (Axial, Coronal, Sagittal)	30-Mid Slices	1.4709	0.7903	0.6667
	3D (Axial, Coronal, Sagittal)	50-Mid Slices	0.4044	0.8710	0.8261
	3D (Axial, Coronal, Sagittal)	All-176 Slices	1.2591	0.7581	0.6512
	3D (Axial, Coronal, Sagittal)	All-176 Except 10-Start & 10-End	0.5081	0.8387	0.7727

**Table 5:** Performance comparison of the proposed transformers and previous studies using fMRI.

Study	Site	Input	No Subjects	Accuracy	F1
MCSE—VGG [17]	NYU	fMRI—Brain Functional Networks	ASD 79; TC 105	77.74%	75.33%
Deep Ensemble Learning—VGG16 [12]	NYU	fMRI—Glass Brain	ASD 79; TC 105	88%	—
3D-CNN (This Study)	NYU	fMRI—Mean Image	ASD 74; TC 98	87%	82%

**Table 6:** Performance comparison of the proposed transformers and previous studies using sMRI.

Study	Site	Input	No Subjects	Accuracy	F1
CNN—ResNet [14]	ABIDE-I	sMRI—Covariance Brain Networks	ASD 518; TC 567	71.81%	68.68%
ViT (This Study)	NYU	sMRI—2D Slices	ASD 79; TC 105	63%	51%
Swin (This Study)	NYU	sMRI—2D Slices	ASD 79; TC 105	58%	36%
MobileNet (This Study)	NYU	sMRI—2D Slices	ASD 79; TC 105	72%	70%
ConvNeXt (This Study)	NYU	sMRI—2D Slices	ASD 79; TC 105	77%	76%

## Data Availability

Data are contained within the article.
